# Monocyte-macrophage sensing of calciprotein particles in CKD-associated vascular calcification: lysosomal stress and inflammasome activation

**DOI:** 10.3389/fimmu.2026.1982842

**Published:** 2026-10-23

**Authors:** Zhiqiang Xu, Yuying Jiang, Kangwei Wang, Zehao Jin

**Affiliations:** 1 The Affiliated Yueqing Hospital, Wenzhou Medical University, Wenzhou, Zhejiang, China; 2 Taizhou Central Hospital (Taizhou University Hospital), Taizhou, Zhejiang, China; 3 Technical University of Munich, Munich, Germany

**Keywords:** calciprotein particles, chronic kidney disease, lysosomal stress, monocytes and macrophages, NLRP3 inflammasome, sterile inflammation, vascular calcification

## Abstract

Chronic kidney disease (CKD) exposes the circulation to sustained calcium-phosphate stress. Calciprotein particles (CPPs) buffer mineral supersaturation and, alongside mineral and bone disorder, uremic toxins, oxidative stress, and inflammation, are an important potential contributor to vascular injury. This mini-review examines how monocytes and macrophages recognize and process CPPs, with emphasis on lysosomal stress and inflammatory responses. Scavenger receptor A contributes to the uptake of mature CPPs in experimental models. In human monocytes studied under high-calcium conditions relevant to rheumatoid arthritis, the calcium-sensing receptor enhances macropinocytosis and CPP uptake. Subsequent responses vary with particle preparation, dose, cell type, and priming. Reported effects include altered lysosomal acidification and proteolysis, and membrane damage in selected models. Genetic and pharmacological studies support the activation of the NLR family pyrin domain containing 3 (NLRP3) inflammasome and the release of caspase-1-dependent interleukin-1β. Interleukin-1α release and necrotic injury can persist independently of this pathway. Separate vascular studies have reported endothelial dysfunction and smooth muscle cell calcification after CPP exposure, but have not established that myeloid CPP sensing mediates these effects in CKD. Particle heterogeneity and non-equivalent assays limit the ability to make comparisons across studies. Interventions that reduce mineral loading or delay CPP maturation affect some particle-related measures, but their effects on clinical vascular outcomes remain uncertain.

## 1 Introduction

Vascular calcification in chronic kidney disease (CKD) is multifactorial. CKD-associated mineral and bone disorder, uremic toxins, oxidative stress, and inflammatory signaling contribute alongside altered vascular cell function and reduced mineral-buffering capacity. Calciprotein particles (CPPs) are an important potential contributor to this broader process; however, current evidence does not establish them as the dominant driver relative to these other factors. CPPs package calcium phosphate into colloidal protein-mineral complexes that can be transported to clearance organs. Experimental studies have also identified the inflammatory and vascular effects of CPP exposure. Therefore, CPP-related measurements and serum calcification propensity provide information about mineral handling, but neither directly measures arterial mineral deposition (1, 2).

The protective and potentially harmful effects of CPPs depend on the experimental setting and the endpoint examined. Mineral stabilization, cellular uptake, cytokine release, and vascular calcification are distinct outcomes.

Studies on CPP maturation and their direct effects on vascular smooth muscle cells (VSMCs) are complemented by experiments on monocytes and macrophages, which participate in particle uptake and systemic clearance. Recent reviews have summarized mineral trafficking and potential interventions (3, 4). This review examines myeloid CPP handling across particle preparations, species, and disease contexts, with particular attention to the relevance and limitations of these models for CKD-associated vascular calcification.

## 2 CPP formation and maturation in CKD

Fetuin-A can stabilize small calcium-phosphate clusters as calciprotein monomers (CPMs), as demonstrated by small-angle neutron scattering experiments (5). Under mineral supersaturation, fetuin-A also supports the formation of small, rounded primary CPPs (CPP1), which contain predominantly amorphous mineral and can mature into larger, more crystalline secondary CPPs (CPP2s) *in vitro* (6). Albumin and other acidic serum proteins enhance fetuin-A-mediated stabilization of the larger particles (7). Live imaging has identified distinct distributions of CPMs, CPP1s, and CPP2s in mice (8). These categories describe stages of mineral organization, but do not capture the full heterogeneity of particles in human blood.

The serum calcification propensity assay measures T50, which is the half-time of CPP1-to-CPP2 conversion after a standardized supraphysiological calcium and phosphate challenge ex vivo. A shorter T50 indicates less resistance to induced crystallization; however, it does not measure native CPP concentration, intravascular maturation time, or vascular calcium content (9). The assay is influenced by mineral concentrations and the availability of inhibitors and buffers, including fetuin-A, magnesium, albumin, and bicarbonate (9, 10). Renal excretory function alone does not explain the variation in T50 (10). A small, controlled feeding study detected postprandial CPM and CPP excursions in adults with and without CKD, consistent with a physiological role in mineral transport (11).

CPP maturation does not produce a uniform relationship between particle type and toxicity. In macrophage experiments, fetuin-A-containing CPPs caused less injury than calcium-matched naked hydroxyapatite, although they still induced cytokine production (12). Endogenous uremic CPPs have heterogeneous protein, lipid, and mineral characteristics that differ from those of synthetic CPP2s (13). Therefore, CPPs have been considered mineral carriers and potential cellular stressors (14). In one *in vitro* model, CPP2s combined with elevated phosphate increased VSMC mineralization while reducing osteoblast mineralization (15).

Sample handling adds another source of variation. Anticoagulants, storage, freeze-thaw cycles, centrifugation, and incubation can alter the particles detected by an assay (16). Zeper and colleagues showed that synthesis conditions affect particle number, diameter, protein composition, and VSMC-calcifying potency (17). Therefore, equal calcium mass does not ensure equivalent particle exposure. T50, hydrodynamic size, centrifuged CPP-associated fetuin-A, and direct particle counts should be reported as separate measurements.

## 3 Myeloid sensing and inflammatory processing of CPPs

### 3.1 Monocyte–macrophage recognition, uptake, and clearance

In mice, injected mature synthetic CPPs accumulate rapidly in Kupffer cells and splenic marginal-zone macrophages. Herrmann and colleagues found reduced CPP uptake in macrophages lacking scavenger receptor A types I and II (SR-A) and reduced circulatory clearance in mice lacking both SR-A and the macrophage receptor with collagenous structure (MARCO) (18). Subsequent comparisons showed preferential CPP1 uptake by liver sinusoidal endothelial cells, whereas macrophages efficiently took up CPP2s. SR-A blockade or deficiency reduced CPP2 uptake but had little effect on CPP1 uptake (19). Thus, SR-A contributes to mature CPP clearance but does not account for all CPP uptake.

The molecular feature recognized by SR-A remains unresolved. CPP preparations differ in their mineral structure and associated proteins, and receptor studies have generally examined a limited set of laboratory preparations (13, 18, 19). These experiments have identified a receptor contribution to uptake but have not established whether recognition depends on fetuin-A, another component of the protein corona, or the exposed mineral.

A separate uptake mechanism has been described in primary human monocytes studied in the context of rheumatoid arthritis (RA). Jäger and colleagues found that extracellular calcium promoted macropinocytosis, CPP uptake, and NLR family pyrin domain containing 3 (NLRP3)-dependent interleukin (IL)-1β release through calcium-sensing receptor (CaSR) signaling; inhibition of the G-protein αq subunit (Gαq) reduced the calcium-induced cellular signaling response (20). The study did not establish direct binding of CPP to CaSR. Instead, CaSR signaling promoted fluid-phase particle uptake. This model featured high extracellular calcium, phosphate-rich conditions, and inflammatory priming; the same mechanism has not been established in circulating CKD monocytes.

Toll-like receptor 4 (TLR4) deficiency reduced CPP-induced cytokine responses, whereas receptor-blocking experiments did not reduce CPP internalization (19). In whole-blood and cell-culture systems, monocytes took up CPP and showed activation responses, including with preparations intended to approximate physiological exposures (21, 22). These studies did not define the receptors responsible for native CPP uptake in patients with CKD.

Live imaging in mice detected renal filtration of CPM-containing material and hepatic uptake of larger CPP (8). Impaired buffering or clearance has been proposed to prolong systemic particle exposure in CKD-associated mineral and bone disorder (23, 24); however, the cited studies did not define a saturation threshold in patients or establish a CKD-specific macrophage clearance defect.

### 3.2 Phagolysosomal processing and lysosomal stress

After uptake, CPPs localize to endolysosomal compartments. Reported responses include altered cathepsin activity, reduced acidification or proteolysis, and membrane damage under certain conditions (20, 25–27). A common sequence of lysosomal events across CPP preparations and myeloid cell types has not been established.

The temporal relationship between particle dissolution, calcium and phosphate transport across the lysosomal membrane, and changes in cytosolic ions remains unresolved in primary CKD myeloid cells. Existing studies have not jointly measured these events or identified the responsible transporters.

Lysosomal responses differ between experimental systems. In the CaSR-supported human monocyte model, CPP uptake increased cathepsin activity and IL-1β release, and cathepsin inhibitors reduced the cytokine response. Acridine orange imaging did not detect lysosomal leakage (20). In contrast, concentrated CPP2s localized to lysosomes and caused membrane damage in lipopolysaccharide (LPS)-primed murine macrophages (25). The latter finding supported membrane injury under those exposure conditions, but did not make lysosomal rupture a universal consequence of CPP uptake.

With sustained extracellular calcium and CPP exposure in the absence of LPS priming, human monocytes showed reduced lysosomal proteolysis and LysoTracker staining, increased expression of lysosome-associated membrane protein 2 (LAMP2), and reduced mechanistic target of rapamycin complex 1 (mTORC1) signaling. Reduced acidification was further supported by LysoSensor measurements in THP-1 cells, a human monocytic cell line (26). Nuclear localization of transcription factor EB (TFEB) and signal transducer and activator of transcription 3 (STAT3) accompanied differentiation into macrophages producing secreted phosphoprotein 1 (SPP1), also known as osteopontin (26). This program was studied using healthy-donor and RA monocytes, not a CKD model. In human renal proximal tubular epithelial HK-2 cells, crystalline CPP increased lysosomal pH, impaired hydrolase activity and autophagic flux, and altered cholesterol distribution without causing detectable membrane permeabilization (27). However, these epithelial-cell measurements cannot substitute for equivalent measurements in macrophages.

CPP exposure also disrupted autophagic processing in endothelial cells and VSMCs, with effects that depended on cell type and particle state (28). That study did not establish impaired macrophage autophagy or efferocytosis in CKD. How these lysosomal and autophagic changes relate to one another remains unresolved in primary CKD myeloid cells.

### 3.3 Inflammasome-dependent and -independent outputs

CPP-induced cytokine responses were observed in early macrophage studies, with lower tumor necrosis factor (TNF) and IL-1β release after CPP exposure than after calcium-matched naked hydroxyapatite exposure (12). At matched calcium content, CPP1s induced more apoptosis-associated speck-like protein containing a caspase recruitment domain (ASC) specks and IL-1β release than CPP2s, whereas CPP2s induced more TNF (19). Live-cell imaging also detected earlier ASC speck formation after CPP1 exposure than CPP2 exposure, whereas CPM-enriched preparations did not induce specks (8). ASC specks indicate inflammasome assembly, but they do not identify the sensor by themselves. Neither comparison directly measured cytosolic calcium changes nor established dissolution kinetics as the cause of the different inflammatory responses.

In calcium/CPP-exposed human monocytes, pharmacological inhibition of NLRP3 or cathepsin B reduced IL-1β release (20). In CPP2-exposed murine macrophages, Anzai and colleagues found that genetic deletion or pharmacological inhibition of NLRP3 or caspase-1 markedly reduced mature IL-1β release. Elevated extracellular potassium levels suppressed IL-1β release, indirectly supporting potassium efflux; the tested antioxidants did not suppress IL-1β release (25). These studies support NLRP3-dependent responses within their respective models, but they do not establish a uniform upstream activation mechanism.

In the murine CPP2 model, IL-1α release and necrotic injury persisted in the absence of NLRP3 or caspase-1. Although gasdermin D (GSDMD) cleavage was detected, deletion of its gene did not prevent IL-1β release or cell death. Acute particle injection also produced IL-1-dependent neutrophil recruitment, with a prominent contribution from IL-1α and the C-X-C motif chemokine ligand 1 (CXCL1)-C-X-C motif chemokine receptor 2 (CXCR2) pathway (25). Therefore, the response cannot be classified as obligatorily GSDMD-dependent pyroptosis.

Priming and inflammasome activation should be distinguished. TLR4-dependent cytokine responses were reported in macrophages, although the IL-1β assays used LPS priming (19). Priming was also used in the acute human calcium/CPP model (20). CPP2s could themselves provide priming and activation signals in the murine experiments of Anzai and colleagues (25). These findings do not establish that native CPPs provide sufficient priming in CKD.

Responses beyond IL-1β release have also been reported. CPP exposure elicited broader cytokine and chemokine responses in human monocyte cultures, with effects varying by particle preparation (22). In the sustained calcium/CPP model without LPS, monocytes differentiated into osteopontin-producing macrophages (26). These studies broaden the range of measured responses, but they do not establish that the cytokine and differentiation programs are independent of NLRP3.

## 4 From myeloid CPP sensing to vascular dysfunction and calcification

Direct vascular cell experiments should be distinguished from evidence of myeloid-mediated injury. Aghagolzadeh and colleagues found that synthetic CPP2s induced oxidative stress, TNF expression and release, and calcification in cultured human VSMCs; silencing TNF or TNF receptor 1 (TNFR1) attenuated calcification, whereas CPP1s did not induce calcification under the tested conditions (29). CPP and extracellular vesicle fractions isolated from patients with stage 5 CKD also promoted VSMC osteogenic responses and calcification (30). The heterogeneity of the isolated fractions limits attribution to a single native CPP subtype. Neither study establishes that a macrophage intermediate is required for the vascular response.

CPP exposure was shown to impair endothelial nitric oxide synthase (eNOS) expression or coupling, increase oxidative stress, and reduce nitric oxide-dependent vascular relaxation in experimental systems (31). Proteomic analysis identified changes in endothelial adhesion, metabolism, and stress-related pathways (32). Conditioned medium from CPP-treated endothelial cells was found to increase the expression of intercellular adhesion molecule 1 (ICAM1) and vascular cell adhesion molecule 1 (VCAM1) and the release of C-C motif chemokine ligand 2 (CCL2), IL-6, and IL-8 in previously untreated human coronary artery endothelial cells (21). Conditioned medium from CPP2-treated endothelial cells was also found to enhance VSMC calcification (33). This supports endothelial paracrine signaling, but does not establish a CPP-activated macrophage-to-VSMC pathway.

Particle size and the exposure system affect vascular responses. Studies have described size-dependent endothelial interactions and CPP-associated calcium overload with mitochondrial dysfunction (34, 35). In isolated mouse aortas, CPP1s, but not CPP2s, increased passive stiffness, most clearly in the abdominal infrarenal region, without detectable medial mineralization. CPP1s also impaired contraction and endothelium-dependent relaxation; 2 weeks of CPP administration to healthy mice did not increase basal aortic stiffness (36). These findings distinguish vascular dysfunction from mineral deposition; they do not establish a temporal progression from one to the other or explain the negative *in vivo* result.

Human evidence is largely observational, with results that vary across assays and cohorts. In predialysis CKD, CPP-associated fetuin-A was associated with aortic stiffness (37). Shorter T50 was associated with aortic stiffness, stiffness progression, and mortality, although the association with mortality was lost after additional adjustment for aortic stiffness (38). In the Chronic Renal Insufficiency Cohort (CRIC) Study, shorter T50 was found to be associated with coronary artery calcification severity and progression, but associations with clinical events were attenuated after adjustment for kidney function and proteinuria (39, 40). In incident hemodialysis patients, T50 and generated CPP2 size were not independently associated with coronary or thoracic aortic calcium or pulse-wave velocity (41). A smaller advanced CKD study found an association between larger generated CPP2, but not T50, and vascular calcification (42). Differences in populations, measurements, and adjustments may contribute to these findings, but their relative effects have not been resolved.

Direct particle counts have been associated with vascular remodeling transcripts in CKD tissues, and CPM concentrations with CKD progression (43, 44). Simultaneous assay comparisons found differing relationships between CPP-related measurements and clinical outcomes (45). These observations do not establish that native CPP uptake by monocytes causes lysosomal stress, inflammasome activation, and subsequent medial calcification. **Figure 1** summarizes the experimental mechanisms and the proposed myeloid-to-vascular link; **Table 1** outlines the models and evidence limitations.

**Figure 1:**
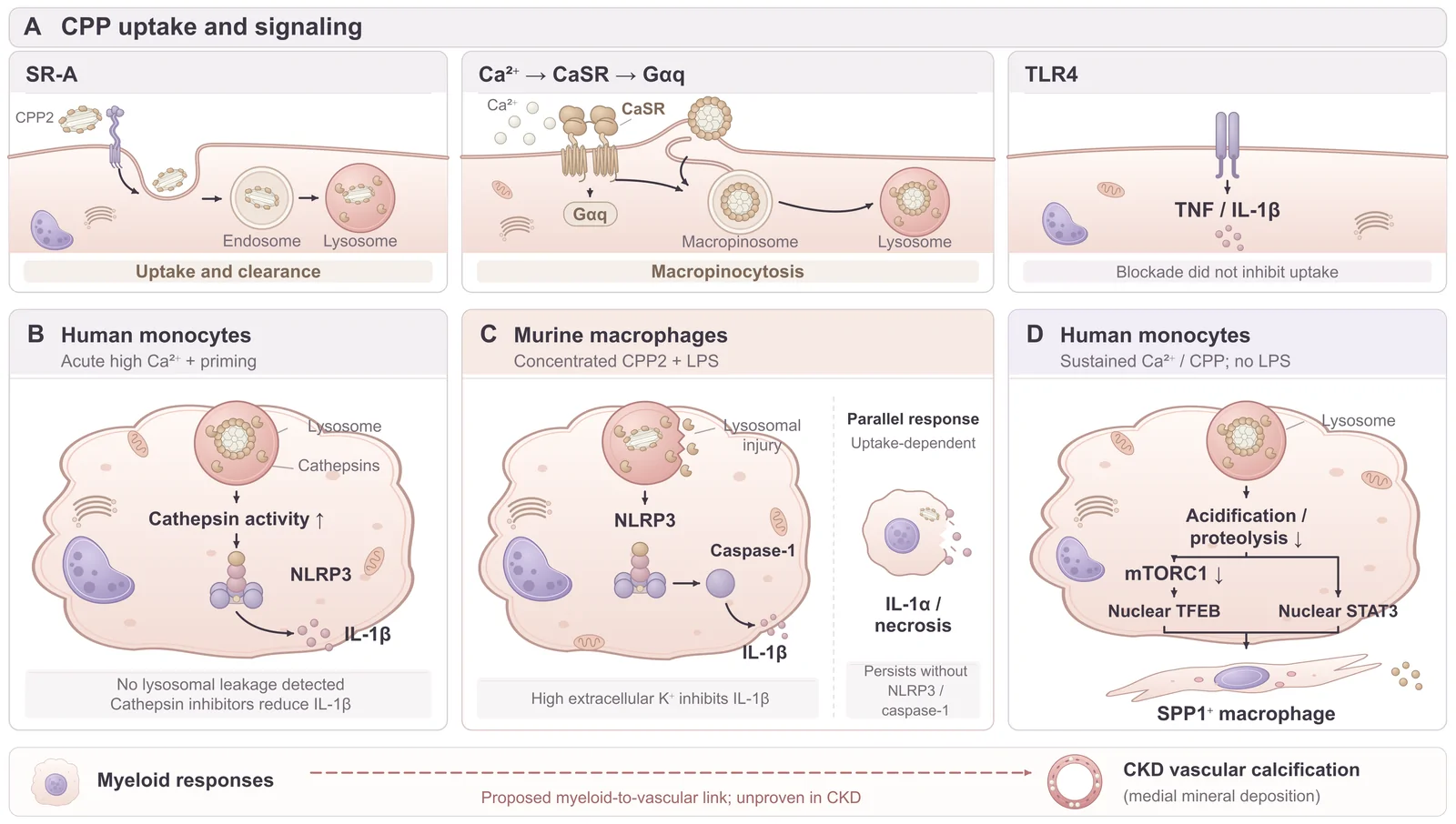
Calciprotein particle uptake and myeloid responses. **(A)** Uptake and receptor signaling. **(B)** Acute inflammatory responses in primed human monocytes. **(C)** Lysosomal injury and inflammatory responses in primed murine macrophages. **(D)** Lysosomal dysfunction and macrophage differentiation in human monocytes without lipopolysaccharide. The solid arrows summarize the findings within each model; the dashed arrow denotes an unproven link between myeloid cells and vascular calcification in chronic kidney disease.

**Table 1:** Key experimental evidence for CPP uptake and myeloid responses.

Study	Particles and model	Uptake/processing	Main responses	Evidence limitations
Herrmann et al., 2012 (18)	Synthetic mature fetuin-A CPPs; mice and tissue macrophages.	SR-A loss reduced macrophage uptake; combined SR-A/MARCO loss impairs circulatory clearance.	Rapid hepatic and splenic macrophage accumulation; reduced clearance in double-deficient mice.	Acute injection; non-CKD; SR-A is not the sole uptake route.
Smith et al., 2013 (12)	Synthetic CPPs; RAW264.7 macrophages.	SR-A type I blockade reduced CPP uptake; other uptake routes remain possible.	TNF and IL-1β induction, with less injury than calcium-matched naked hydroxyapatite.	Most functional experiments used synthetic CPPs in a mouse cell line.
Köppert et al., 2018 (19)	Synthetic CPP1s/CPP2s; mice, liver endothelial cells and macrophages.	CPP2 uptake partly SR-A-dependent; CPP1 favored liver endothelial uptake.	CPP1s favored ASC specks/IL-1β; CPP2 favored TNF. TLR4 deficiency reduced cytokines; blockade did not inhibit uptake.	Acute models; synthetic particles; calcium-normalized comparisons.
Jäger et al., 2020 (20)	Human monocytes; high calcium/phosphate with inflammatory priming.	CaSR promoted calcium-dependent macropinocytosis; Gαq signaling was detected.	Increased cathepsin activity and NLRP3-dependent IL-1β; no lysosomal leakage detected.	RA/high-calcium context; direct CPP-CaSR binding was not established.
Anzai et al., 2021 (25)	Concentrated synthetic CPP2s; LPS-primed murine macrophages.	Phagocytosis required; lysosomal injury detected; receptor not identified.	NLRP3/caspase-1-dependent IL-1β; IL-1α release and necrosis persist without NLRP3 or caspase-1.	High-dose, non-CKD model; potassium-efflux evidence is indirect.
Koeppert et al., 2021 (8)	CPM-enriched material and CPP1s/CPP2s; live mice and macrophages.	Renal filtration of CPM-enriched material; hepatic retention of larger CPPs.	CPP1s induced earlier ASC specks than CPP2s; CPM-enriched preparations did not.	CPM fraction largely free of fetuin-A; ASC specks do not identify NLRP3.
Murthy et al., 2022 (26)	Human monocytes; sustained calcium/CPPs without LPS; THP-1 validation.	Reduced lysosomal proteolysis; dye-based evidence of impaired acidification.	Reduced mTORC1 signaling; nuclear TFEB/STAT3 accompanied SPP1-producing macrophage differentiation.	Healthy-donor/RA cells; no CKD or vascular endpoint.

Findings are model-specific and do not establish a causal myeloid pathway in CKD. Abbreviations are defined in the Abbreviations section.

## 5 Therapeutic evidence

Interventions that alter mineral loading or particle maturation have been examined experimentally and in small clinical trials. Magnesium delayed CPP1-to-CPP2 conversion and prevented high-phosphate-induced VSMC calcification *in vitro*; however, it did not prevent calcification when added with preformed CPP2s (46). Increasing dialysate magnesium improved T50 in a randomized trial (47). This trial demonstrated a change in an ex vivo calcification propensity measure, rather than a reduction in clinical vascular events.

Findings on phosphate binders are mixed. In the Sevelamer Versus Calcium to Reduce Fetuin-A-Containing Calciprotein Particles in Dialysis (SCaRF) trial, sevelamer reduced CPP1s, aortic pulse wave velocity, and IL-8 relative to calcium carbonate, without a significant reduction in CPP2s (48). Compared with binder washout, short-term high-dose sucroferric oxyhydroxide reduced endogenous CPP1s and CPP2s and attenuated the procalcific and endothelial-activating effects of patient serum *in vitro* (49). Lanthanum carbonate did not significantly reduce CPP1s or CPP2s over 96 weeks in CKD stages 3b-4 (50). In a separate trial involving stage 3b-4 CKD patients, sevelamer carbonate did not significantly change T50 over 12 weeks (51). These trials do not establish a uniform phosphate binder effect on CPP-related measures or explain the differences between treatments.

## 6 Future perspectives

One hypothesis is that prolonged CPP exposure links mineral transport to myeloid inflammation when particle processing is insufficient. Different rates of dissolution and particle persistence may produce distinct lysosomal stresses. However, this explanation requires validation across experimental systems. A clearance-saturation threshold in patients and a causal sequence from myeloid CPP uptake to medial calcification also remain to be established.

Future studies could test this model through lineage-restricted perturbation of uptake or inflammasome signaling in CKD models and by exposing primary myeloid cells to freshly isolated patient CPPs. Particle number, mineral content, and surface area should be reported alongside serial measurements of lysosomal pH, membrane integrity, and intracellular ions. These measurements would help distinguish a transport defect from substrate overload and determine whether the myeloid response precedes vascular injury.

Reducing inflammatory signaling while preserving mineral clearance may offer advantages over broadly inhibiting CPP uptake, but this remains a therapeutic hypothesis. Combined SR-A/MARCO deficiency reduces circulatory CPP clearance in mice, and NLRP3 blockade leaves IL-1α release and necrotic injury detectable in the tested macrophage model (18, 25). Clearance, cytokine release, and vascular outcomes should therefore be assessed separately; the efficacy and safety of these approaches in CKD remain unestablished. Changes in T50 or particle counts should be evaluated alongside vascular endpoints rather than assumed to predict clinical benefit.

## 7 Conclusions

CPPs participate in mineral buffering and can induce myeloid and vascular cell responses under defined experimental conditions. Receptor perturbation, lysosomal measurements, and inflammasome studies support individual mechanisms, but their contribution to CKD-associated vascular calcification has not been established as a continuous causal pathway. CPPs should therefore be considered alongside other contributors to vascular injury. Disease-relevant mechanistic studies and vascular outcome trials are needed to determine whether modifying CPP handling improves vascular health.
